# Long-term change in body composition following intentional weight loss and its effect on physical function

**DOI:** 10.1038/s41366-025-01997-x

**Published:** 2025-12-23

**Authors:** Kacey Chae, Xiaoyan Leng, Rebecca H. Neiberg, George A. Bray, Karen C. Johnson, James O. Hill, John M. Jakicic, Ariana M. Chao, Kristen M. Beavers, Henry J. Pownall, Stephen B. Kritchevsky, Denise K. Houston

**Affiliations:** 1 Department of Medicine, Johns Hopkins University School of Medicine, Baltimore, MD USA; 2 Department of Biostatistics and Data Science, Wake Forest University School of Medicine, Winston-Salem, NC USA; 3 Pennington Biomedical Research Center, Louisiana State University, Baton Rouge, LA USA; 4 Department of Preventive Medicine, University of Tennessee Health Science Center, Memphis, TN USA; 5 Department of Nutrition Sciences, University of Alabama at Birmingham, Birmingham, AL USA; 6 Department of Internal Medicine, University of Kansas Medical Center, Kansas City, KS USA; 7 Johns Hopkins School of Nursing, Baltimore, MD USA; 8 Department of Internal Medicine, Wake Forest University School of Medicine, Winston-Salem, NC USA; 9 Department of Cardiology, Houston Methodist Research Institute and Baylor College of Medicine, Houston, TX USA

**Keywords:** Risk factors, Geriatrics

## Abstract

**Background/Objectives:**

A concern with intentional weight loss among middle-aged and older adults with obesity and type 2 diabetes mellitus (T2DM) is the loss of lean mass, which may lead to declining physical function. However, the association between changes in body composition with intentional weight loss and physical function over the long-term is unknown. Thus, we examined the association between changes in body composition and physical function 8 years following an intensive lifestyle intervention (ILI).

**Subjects/Methods:**

We conducted a secondary analysis of participants at the Baton Rouge site within the Look AHEAD study. Participants (n = 220) were middle-aged and older adults with overweight/obesity and T2DM randomized to an ILI or diabetes support and education (DSE). Body composition was measured by dual-energy X-ray absorptiometry at baseline and Year 8. Physical performance (expanded Short Physical Performance Battery [SPPB_exp_], 20- and 400-m walk) and strength (grip and knee extensor) were assessed at Year 8.

**Results:**

Percent change (mean ± SD) from baseline in weight, fat and lean mass over 8 years were −4.0 ± 7.3%, 0.2 ± 12.5% and −6.5 ± 5.3% in ILI and −3.0 ± 9.7%, 1.2 ± 17.1% and −5.8 ± 6.6% in DSE, respectively. ILI had better SPPB_exp_ scores and faster gait speed than DSE at 8-year follow-up (p < 0.05). Increases in fat mass were associated with worse SPPB_exp_ scores in ILI and DSE (p = 0.03) and with slower gait speed in DSE (p = 0.01). Decreases in lean mass were associated with weaker grip strength in ILI (p = 0.04) and knee extensor strength in ILI and DSE (p < 0.05). There were no significant interactions by intervention group.

**Conclusions:**

Although the overall intervention effect on physical function was positive, increases in fat mass were associated with poorer physical performance while lean mass loss was associated with weaker strength 8 years post-randomization. Findings highlight the importance of minimizing fat mass gain/regain and loss of lean mass during intentional weight loss.

## Introduction

Obesity, which impacts approximately 45% of middle-aged adults and 40% of older adults [1], is associated with declines in mobility and physical function [2, 3]. Poor physical function is a predictor of disability and mortality as well as greater healthcare costs [4, 5]. Furthermore, type 2 diabetes mellitus (T2DM) accelerates declines in lean mass, worsens muscle strength/quality, and increases risk of physical disability [6]. Therefore, adults with obesity and T2DM are at high risk for functional decline, underscoring the importance of identifying strategies that optimize physical function in this population.

Over the short-term, physical function can be improved through lifestyle changes that promote weight loss. In older adults with overweight/obesity, current evidence suggests that participation in weight loss and exercise interventions lasting up to 18 months improves performance-based physical function measures [7–14]. Studies examining the long-term effect of intensive lifestyle intervention—consisting of moderate caloric restriction, increased physical activity, and behavior modification—in middle-aged and older adults with obesity and T2DM also showed that those randomized to the intensive lifestyle intervention had better performance-based physical function compared to the control group [15, 16].

However, recommending intentional weight loss in older adults and individuals with T2DM at risk for frailty remains controversial, which, in part, stems from the concern about loss of lean mass with intentional weight loss [17], leading to impaired physical function [18–20]. Several prior studies show that short-term changes in lean mass accompanying intentional weight loss were not associated with declines in strength or physical performance [21–24]. Rather, fat mass loss was significantly associated with improvements in physical performance [25], possibly due to improvement in muscle quality from less fat infiltration into the skeletal muscle tissue [26]. However, on the background of the cumulative effects of age-related declines in lean mass, the association between long-term changes in body composition following intentional weight loss and physical function is unknown, particularly among middle-aged and older adults with obesity and T2DM.

Thus, the objective of this study was to examine the association between long-term changes in body composition following intentional weight loss (Intensive Lifestyle Intervention, ILI) and a comparison group (Diabetes Support and Education, DSE) over 8 years and physical function. We hypothesized that, compared to the DSE group, increases in fat mass and decreases in lean mass over 8 years in ILI would be associated with poorer physical function 8 years after randomization. We also explored whether the associations between change in body composition and physical function differed by age.

## Subjects and methods

### Study setting and population

The design and methods of the Action for Health in Diabetes (Look AHEAD) trial have been published previously ([27]; NCT00017953, NCT0141009). In brief, Look AHEAD recruited individuals with T2DM who were 45–76 years of age and had a body mass index ≥25 kg/m^2^ (or ≥27 kg/m^2^ in participants on insulin), HbA1_c_ < 11%, systolic blood pressure <160 mmHg, diastolic blood pressure <100 mmHg, and triglycerides <600 mg/dl at 16 clinical sites across the USA. These individuals underwent a maximal graded exercise test to ensure that exercise could be safely prescribed and completed two weeks of self-monitoring prior to randomization. Data for this analysis are from the Baton Rouge site, the only Look AHEAD site that was part of the dual-energy X-ray absorptiometry (DXA) sub-study and the Look AHEAD Movement and Memory (Look AHEAD M&M) ancillary study.

### Ethics approval and consent to participate

Protocols were approved by the Wake Forest University School of Medicine institutional review board (IRB # BG99-042) and the local institutional review board at the Baton Rouge Look AHEAD site (Louisiana State University). All procedures were performed in accordance to the approved protocols and all participants provided written informed consent.

### Interventions

At enrollment into the Look AHEAD trial, participants were randomly assigned to the ILI or DSE group. ILI included diet modification and increased physical activity and was designed to achieve and maintain the loss of at least 7% of initial weight [28]. ILI participants were assigned a calorie goal (1200–1800 kcals/day based on initial weight), with <30% of total calories from fat and a minimum of 15% of total calories from protein. The physical activity goal was ≥175 minutes of unsupervised, moderately intense physical activity per week focused on activities similar in intensity to brisk walking. During Year 1, ILI participants were seen weekly for the first 6 months and 3 times per month for the next 6 months, with a combination of group and individual sessions. During Years 2–4, participants were seen individually at least once per month and had a minimum of one additional contact by phone, mail or email per month. During Years 5 + , participants were encouraged to continue individual monthly sessions, and annual campaigns were used to promote adherence.

DSE participants were invited to three group sessions focused on diet, physical activity, or social support each year for the first 4 years and one session annually thereafter [29]. Information on behavioral strategies was not presented, and participants were not weighed at these sessions.

### Physical function

The Look AHEAD M&M ancillary study assessed performance-based physical function at four Look AHEAD clinic sites at the Year 8 or 9 clinic visit from September 2009 through June 2012. Only Look AHEAD participants who were currently active (i.e., had not died, been lost to follow-up, or refused further Look AHEAD activity) and who provided separate informed consent were eligible to enroll in the ancillary study. Certified clinic staff masked to intervention assignment conducted all physical function measures.

The Short Physical Performance Battery (SPPB) was administered to assess lower extremity physical function [30]. The SPPB consists of standing balance tasks (side-by-side, semi- and full-tandem stands for 10 seconds each), a 4-m walk to assess usual gait speed, and time to complete 5 repeated chair stands. Each of the three performance measures is assigned a score ranging from 0 (inability to perform the task) to 4 (the highest level of performance) and summed to create an SPPB score ranging from 0 to 12 (best). The SPPB was modestly expanded (SPPB_exp_) to minimize ceiling effects of the SPPB when used in well-functioning populations such that the holding time of the standing balance tasks was increased to 30 seconds and a single leg stand was added [31]. The SPPB_exp_ component scores were calculated as the ratio of observed performance to the best possible performance and summed to provide a continuous score ranging from 0 to 3, with higher scores indicative of better performance.

Usual walking speed over 20 meters and walking endurance over 400 meters were measured [32]. The course was 20-m long marked by cones at each end. Participants were instructed to walk at their usual pace and time to complete the first 20-m and the 400-m walk was recorded. Gait speed over 400 meters was calculated for those participants who completed the walk.

Grip strength (kg) was measured twice in each hand using an isometric Hydraulic Hand Dynamometer (Jamar, Bolingbrook, IL, USA) with the participant in a seated position and their arm resting on a table with the elbow bent at a 90-degree angle. The mean grip strength from two trials for the stronger hand was used in the analyses. Maximum knee extensor strength (kg; one repetition maximum) was assessed on a Nautilus One™ Leg Extension machine. The right leg was tested unless there was a contraindication (e.g., prior knee surgery). If participants experienced knee pain during the test and there were no contraindications to test the other leg, then the other leg was tested.

### Weight and body composition

Clinic staff masked to intervention assignment collected annual measures of weight throughout the trial using a digital scale. Body composition (total body lean and fat mass) was measured by DXA (Hologic, QDR-4500A) at four Look AHEAD sites. Lean mass (i.e., lean soft tissue) was calculated as the difference between fat-free mass and bone mineral content. The coefficient of variation for fat mass was 1.5% in individuals with a BMI < 30 kg/m^2^ and a BMI ≥ 30 kg/m^2^ [33]. The coefficient of variation for lean mass was 0.45% in individuals with a BMI < 30 kg/m^2^ and 0.80% in individuals with a BMI ≥ 30 kg/m^2^ [33]. Longitudinal performance was monitored with regular scanning of a spine phantom and a whole-body phantom, and longitudinal corrections were applied to participant body composition results based on the whole-body phantom. Whole body scan results were corrected for underestimation of fat mass using Hologic software [34]. Participants weighing more than 300 pounds were not scanned due to DXA scanner weight limits. Percent changes in fat and lean mass were calculated using DXA data from baseline, Year 1, and Year 8. Percent change rather than absolute change was used to ensure greater body composition changes in individuals with higher baseline weight would carry similar weight as smaller changes in individuals with lower weight.

### Baseline assessment of potential risk factors for physical function

Self-reported characteristics and conditions were assessed using standardized questionnaires at baseline. Participants brought current prescription medications to the baseline visit. The Short Form-36 Health Survey (SF-36) was used as a measure of health status [35]. The SF-36 measures 8 health domains, including general health, physical functioning, and bodily pain, with domain subscale scores ranging from 0 to 100 (higher scores indicating better functioning or well-being). The Beck Depression Inventory (BDI) was used to measure depressive symptom burden [36]. A BDI score ≥10 was used as a marker for symptoms of mild to moderate depressed mood. Height was measured in duplicate using a stadiometer. A maximal graded exercise test was administered at baseline and cardiorespiratory fitness estimated in metabolic equivalents (METs). Blood specimens were collected after a 12-hour fast and were analyzed by the Central Biochemistry Laboratory (Northwest Lipid Research Laboratories, University of Washington, Seattle, WA, USA) using standardized laboratory procedures for measuring HbA1_c_.

### Statistical analysis

Initial analyses involved descriptive statistics. Comparisons of characteristics between groups were analyzed using chi-square tests for proportions and t-tests for continuous variables. Multiple regression models were used to examine associations between percent change in fat or lean mass and the Year 8 (or Year 9) values of performance-based physical function (SPPB and SPPB_exp_ score, 20-m and 400-m gait speed, and grip and knee extensor strength) stratified by intervention assignment. The models were adjusted for the following variables: sex, race/ethnicity, education level, year of visit when physical function was assessed, and baseline age, BMI, HbA_1c_, insulin use, diabetes duration, hypertension status, prior CVD, depressive symptoms, smoking status, cardiorespiratory fitness, and SF-36 Physical Functioning and Bodily Pain subscales. Percent change in lean mass (for the independent variable percent change in fat mass) or percent change in fat (for the independent variable percent change in lean mass) were also added as covariates in a separate model. Because of the higher risk of functional limitations among older adults, the effect of change in body composition stratified by baseline age ( < 60 vs. ≥ 60 years) was also examined. All analyses were performed in SAS 9.4 (Cary, NC, USA) and significance level was set at 0.05.

## Results

The analytic sample included Look AHEAD participants at the Baton Rouge clinic who participated in both the Look AHEAD M&M ancillary study and the DXA sub-study. The Baton Rouge clinic enrolled 338 participants into the Look AHEAD trial. When Look AHEAD M&M enrollment started, 3 of the original participants had withdrawn from Look AHEAD, 19 had died, and 1 was lost to follow-up, leaving 315 participants who attended a Year 8 (or Year 9) visit during the Look AHEAD M&M enrollment period. Of these, 281 (89%) consented to enroll in the Look AHEAD M&M ancillary study, of which 242 were seen in the clinic. Another 22 participants were missing DXA data at baseline and/or Year 8 resulting in 220 participants with both performance-based physical function and DXA data, 65% of the original Baton Rouge cohort (Supplementary Fig. S1).

Compared to the original Look AHEAD cohort at the Baton Rouge clinic, participants in the Look AHEAD M&M ancillary study and DXA sub-study who were included in these analyses had a lower baseline BMI (34.9 vs. 37.0 kg/m^2^, p = 0.006), had higher baseline cardiorespiratory fitness (7.6 vs. 7.0 METs, p = 0.007) and SF-36 General Health scores (47.0 vs. 44.8, p = 0.03), but did not differ by any other risk factors for physical function including baseline age or SF-36 Physical Functioning scores nor was there a difference in the distribution of intervention assignment between enrollees and non-enrollees. In the analytic sample, the baseline characteristics were balanced between intervention groups (Table 1), except that a greater percentage of ILI participants had prior cardiovascular disease (22.4% ILI vs. 11.5% DSE, p = 0.03) and higher cardiorespiratory fitness ( ≥ 7.5 METs; 57.0% ILI vs. 43.4% DSE (p = 0.04)) at baseline compared to DSE participants.

**Table 1:** Characteristics at the time of enrollment among participants at the Baton Rouge Look AHEAD clinic site who participated in the dual-energy X-ray absorptiometry sub-study and the Look AHEAD Movement and Memory ancillary study.

Characteristics	Intensive Lifestyle Intervention (ILI) (n = 107)	Diabetes Support and Education (DSE) (n = 113)	p-value
Age (yrs), mean (SD)	58.9 (7.7)	58.9 (6.7)	0.97
≥ 60 yrs, N (%)	50 (46.7)	50 (44.2)	0.71
Female, N (%)	59 (55.1)	67 (59.3)	0.53
Race/Ethnicity, N (%)			
African-American	17 (15.9)	24 (21.2)	0.57
Non-Hispanic White	84 (78.5)	84 (74.3)	
Other/Multiple	6 (5.6)	5 (4.4)	
Education, N (%)			
High school or less	14 (13.1)	24 (21.2)	0.20
Post high school	47 (43.9)	51 (45.1)	
College/Graduate	44 (41.1)	34 (30.1)	
Other	2 (1.9)	4 (3.5)	
Body Mass Index (kg/m^2^), mean (SD)	34.6 (5.4)	35.3 (5.1)	0.34
≥ 30 kg/m^2^, N (%)	82 (76.6)	94 (83.2)	0.22
Total fat mass (kg), mean (SD)	39.5 (10.3)	40.8 (10.5)	0.34
Total fat mass (%), mean (SD)	40.2 (7.2)	41.3 (7.5)	0.27
Total lean mass (kg), mean (SD)	55.7 (9.7)	55.4 (11.6)	0.89
Total lean mass (%), mean (SD)	57.2 (6.9)	56.2 (7.3)	0.26
HbA1c (%), N (%)			
< 7.0%	65 (60.7)	58 (51.3)	0.35
7.0-8.9%	34 (31.8)	46 (40.7)	
9.0-11.0%	8 (7.5)	9 (8.0)	
Insulin use, N (% yes)	17 (15.9)	16 (14.2)	0.72
Diabetes duration ≥ 5 yrs, N (%)	49 (45.8)	65 (57.5)	0.08
Hypertension, N (% yes)	90 (84.1)	93 (82.3)	0.72
Prior cardiovascular disease, N (% yes)	24 (22.4)	13 (11.5)	0.03
Depressive symptoms, N (%)	11 (10.3)	9 (8.0)	0.55
Smoking status, N (%)			
Never	58 (54.2)	73 (64.6)	0.10
Former	44 (41.1)	39 (34.5)	
Current	5 (4.7)	1 (0.9)	
Cardiorespiratory fitness (METs), mean (SD)	7.7 (1.9)	7.4 (1.9)	0.22
≥ 7.5 METs, N (%)	61 (57.0)	49 (43.4)	0.04
SF-36, mean (SD)			
General Health score	47.1 (9.4)	47.0 (8.9)	0.92
Physical Functioning score	47.9 (8.6)	46.6 (9.1)	0.28
Bodily Pain score	49.2 (9.2)	48.6 (9.0)	0.61

*HbA1c* Glycated hemoglobin, *METs* Metabolic Equivalents, *SF-36* Short Form-36 Health Survey.

**Fig. 1:**
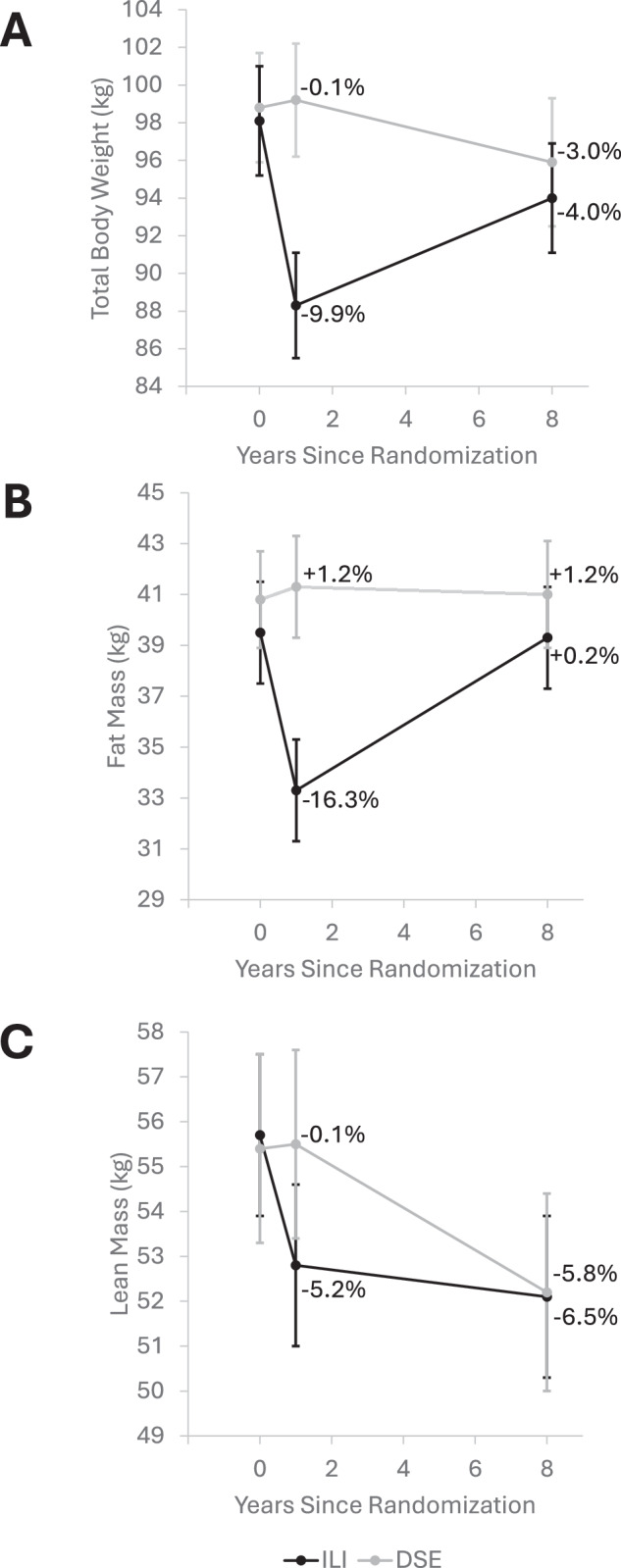
Change in body weight and body composition from baseline to year 8 among participants at the Baton Rouge Look AHEAD clinic site who participated in the dual-energy X-ray absorptiometry sub-study and the Look AHEAD Movement and Memory ancillary study by intervention assignment. Changes in total body weight* (**A**), fat mass (**B**), and lean mass (**C**). *ILI* Intensive Lifestyle Intervention, *DSE* Diabetes Support and Education. Error bars represent 95% confidence intervals of mean total body weight, fat mass, and lean mass at baseline, Year 1, and Year 8. *Total body weight measured via digital scale.

Figure 1 shows the changes in total body weight, fat mass, and lean mass over 8 years. Although ILI participants had significantly greater weight loss than DSE participants from baseline to Year 1 (mean (SD): −9.9% (5.7%) vs. −0.1% (4.1%), respectively; p < 0.001), weight change from baseline to Year 8 did not differ significantly between intervention groups (mean (SD) −4.0% (7.3%) vs. −3.0% (9.7%), respectively; p = 0.38). Those in the ILI group also initially lost a significant amount of fat mass (mean (SD): −16.3% (11.6%), p < 0.001) from baseline to Year 1 but subsequently regained fat mass (p = 0.90 for overall change from baseline), and both ILI and DSE lost lean mass (p < 0.001 from baseline) such that the percent change in lean (mean (SD): −6.5% (5.3) vs. −5.8% (6.6), respectively, p = 0.37) and fat mass (mean (SD): 0.2% (12.5) vs. 1.2% (17.1), respectively, p = 0.60) over 8 years did not differ by intervention group.

The physical function data were collected an average (range) of 8.0 (7.7–9.1) years after randomization. Table 2 shows the distribution of physical performance and strength grouped by intervention assignment. ILI participants had significantly higher SPPB scores, SPPB_exp_ scores and faster 20- and 400-m gait speed than the DSE participants. There were no significant differences in grip or knee extensor strength by intervention group.

**Table 2:** Average physical function at 8-year follow-up among participants at the Baton Rouge Look AHEAD clinic site who participated in the dual-energy X-ray absorptiometry sub-study and the Look AHEAD Movement and Memory ancillary study grouped by intervention assignment.

Physical Function Measure	Intensive Lifestyle Intervention (ILI)		Diabetes Support and Education (DSE)		p-value^a^	p-value^b^
	N	Mean (SD)	N	Mean (SD)		
SPPB score (range 0–12)	102	10.35 (1.60)	108	9.67 (2.09)	0.008	0.03
SPPB_exp_ score (range 0–3)	107	1.70 (0.38)	113	1.53 (0.41)	0.001	0.001
20-meter gait speed (m/sec)	106	1.19 (0.17)	106	1.14 (0.19)	0.02	0.03
400-meter gait speed (m/sec)	97	1.11 (0.15)	100	1.04 (0.18)	0.003	0.002
Grip strength (kg)	97	30.0 (9.3)	98	29.6 (10.5)	0.78	0.43
Knee extensor strength (maximum weight lifted; kg)	85	22.7 (12.2)	80	22.7 (13.5)	0.99	0.45

*SPPB* Short Physical Performance Battery, *SPPB*_exp_ Expanded Short Physical Performance Battery.

^a^Unadjusted.

^b^Adjusted for sex, race/ethnicity, education, year of visit when physical function was assessed, and baseline age, BMI, HbA1c, insulin use, diabetes duration, hypertension status, prior CVD, depressive symptoms, smoking, cardiorespiratory fitness, and SF-36 Physical Functioning and Bodily Pain Subscale.

Tables 3 and 4 show the associations between percent change in body composition over 8 years and physical performance and strength by intervention assignment. Increases in fat mass (Table 3) were associated with worse SPPB_exp_ scores in the ILI and DSE groups (p = 0.03 for both ILI and DSE groups) and with slower 20-m gait speed in the DSE group (p = 0.01). The associations were similar when further adjusted for change in lean mass (Supplementary Table S1). Decreases in lean mass (Table 4) were associated with weaker grip strength, with associations appearing stronger in the ILI group (p = 0.04) than in the DSE group (p = 0.09). Decreases in lean mass were also associated with weaker knee extensor strength in both ILI (p = 0.001) and DSE groups (p = 0.02). When adjusted for change in fat mass, only knee extensor strength in the ILI group remained associated with change in lean mass (Supplementary Table S2). There were no significant interactions by intervention assignment.

**Table 3:** Mean differences in physical function measures by percent change in fat mass over 8 years in intensive lifestyle intervention (ILI) and diabetes support and education (DSE) participants at the Baton Rouge Look AHEAD clinic site who participated in the dual-energy X-ray absorptiometry sub-study and the Look AHEAD Movement and Memory ancillary study.

Physical Function Measure^a^	Intensive Lifestyle Intervention (ILI)		Diabetes Support and Education (DSE)		ILI and DSE combined: Physical Function by intervention assignment interaction p-value
	Beta (SE) for 1% increase in % Fat Mass	p-value	Beta (SE) for 1% increase in % Fat Mass	p-value	
SPPB score (range 0–12)	0.004 (0.012)	0.74	−0.014 (0.011)	0.20	0.29
SPPB_exp_ score (range 0–3)	−0.005 (0.002)	0.03	−0.004 (0.002)	0.03	0.87
20-meter gait speed (m/sec)	−0.002 (0.001)	0.20	−0.003 (0.001)	0.01	0.61
400-meter gait speed (m/sec)	−0.001 (0.001)	0.42	−0.002 (0.001)	0.07	0.63
Grip strength (kg)	0.060 (0.062)	0.34	−0.009 (0.046)	0.84	0.25
Knee extensor strength(maximum weight lifted; kg)	0.033 (0.082)	0.69	0.127 (0.064)	0.05	0.62

*SPPB* Short Physical Performance Battery, *SPPB*_exp_ Expanded Short Physical Performance Battery.

^a^Model adjusted for sex, race/ethnicity, education, year of visit when physical function was assessed, and baseline age, BMI, HbA1c, insulin use, diabetes duration, hypertension status, prior CVD, depressive symptoms, smoking, cardiorespiratory fitness, SF-36 Physical Functioning and Bodily Pain Subscale.

**Table 4:** Mean differences in physical function measures by percent change in lean mass over 8 years in intensive lifestyle intervention (ILI) and diabetes support and education (DSE) participants at the Baton Rouge Look AHEAD clinic site who participated in the dual-energy X-ray absorptiometry sub-study and the Look AHEAD Movement and Memory ancillary study.

Physical Function Measure^a^	Intensive Lifestyle Intervention (ILI)		Diabetes Support and Education (DSE)		ILI and DSE combined: Physical Function by intervention assignment interaction p-value
	Beta (SE) for 1% decrease in % Lean Mass	p-value	Beta (SE) for 1% decrease in % Lean Mass	p-value	
SPPB score (range 0–12)	−0.032 (0.029)	0.27	0.021 (0.030)	0.49	0.18
SPPB_exp_ score (range 0–3)	0.004 (0.006)	0.56	0.002 (0.005)	0.76	0.69
20-meter gait speed (m/sec)	0.001 (0.003)	0.82	0.005 (0.003)	0.09	0.17
400-meter gait speed (m/sec)	0.002 (0.003)	0.44	0.002 (0.003)	0.53	0.16
Grip strength (kg)	−0.325 (0.153)	0.04	−0.199 (0.116)	0.09	0.57
Knee extensor strength (maximum weight lifted; kg)	−0.495 (0.179)	0.008	−0.432 (0.179)	0.02	0.28

*SPPB* Short Physical Performance Battery, *SPPB*_exp_ Expanded Short Physical Performance Battery.

^a^Model adjusted for sex, race/ethnicity, education, year of visit when physical function was assessed, and baseline age, BMI, HbA1c, insulin use, diabetes duration, hypertension status, prior CVD, depressive symptoms, smoking, cardiorespiratory fitness, SF-36 Physical Functioning and Bodily Pain Subscale.

The effect of percent change in body composition over 8 years on physical function appeared to differ by baseline age for several of the physical performance measures and for knee extensor strength (p-values for interaction terms, p < 0.05). Thus, we examined the associations between percent change in body composition and physical performance and strength stratified by age and intervention assignment (Supplementary Table S3). In the ILI group, increases in fat mass were associated with worse SPPB_exp_ scores (p = 0.001) and slower 20-m gait speed (p = 0.006) among those aged ≥60 years but not among those <60 years at randomization (both interaction p-values < 0.01). In the DSE group, decreases in lean mass were associated with lower knee extensor strength (p < 0.001) among those aged <60 but not among those ≥60 years at randomization (interaction p-value = 0.03).

## Discussion

Among middle-aged and older adults with overweight/obesity and T2DM, an intensive lifestyle intervention designed to achieve weight loss through caloric restriction and increased physical activity was associated with overall better physical performance 8 years after randomization, compared to the comparison group. The observed between-group differences in physical performance measures at 8-years were considered clinically significant, based on minimum clinically meaningful differences of 0.5 points for SPPB and 0.05 m/sec for gait speed [37]. However, when examining body composition changes and physical function, increases in fat mass over 8 years were associated with worse physical performance and decreases in lean mass were associated with weaker strength in both the intensive lifestyle intervention and the comparison group. To our knowledge, this is the first study assessing the long-term impact of an intensive lifestyle intervention on body composition changes and physical performance and strength among middle-aged and older adults with overweight/obesity and T2DM, a population at high risk for functional decline. Most trials assessing the benefits of lifestyle-based weight loss interventions on physical performance and strength in middle-aged and older adults with overweight/obesity have been of shorter duration (12 to 18 months) [7–14]. These studies showed that physical performance and strength improved in the short-term in older adults with obesity despite the loss of lean mass [8, 10, 11, 13, 14, 24]. Furthermore, lifestyle-based weight loss interventions led to a preferential loss of fat mass [8, 13, 24], which may be a driver of the improvement in physical performance [25].

Previous Look AHEAD studies showed that middle-aged and older adults with overweight/obesity and T2DM who were randomized to an intensive lifestyle intervention had significantly better self-reported physical function [38] and objectively measured physical performance [15] 8 years after randomization compared to the comparison group. The current study adds to the literature by examining how body composition changes associated with an intensive lifestyle intervention and aging impact physical function in the long-term. Our findings are consistent with those observed in the Health, Aging, and Body Composition Cohort Study showing that excess fat mass contributes to poor physical performance in older adults [39]. A possible causal mechanism involves fat infiltration into the skeletal muscle tissue, leading to poor muscle quality [26, 39]. Furthermore, muscle mass is a contributor of muscle strength, which helps explain the association between the loss of lean mass—of which skeletal muscle is a component—and muscle strength [40]. Notably, almost all of the weight regain at Year 8 in the intervention group was fat mass while lean mass continued to decline, consistent with previous studies assessing changes in body composition in individuals with T2DM [17, 41]. Prior work in Look AHEAD showed that weight regain and weight cycling following weight loss in the intervention group was associated with worse physical function in women and weaker strength in men [42]. Our study provides further evidence of the harms of weight regain and weight cycling on body composition and its potential impact on physical function.

Concerns regarding the functional consequences of the loss of lean mass have deterred some clinicians from recommending weight loss in older adults [43, 44]. In this study, the amount of lean mass lost in both the ILI and DSE groups were at the higher end of what would be expected with aging alone, possibly due to the compounded impact of T2DM, which accelerates declines in lean mass and worsens muscle strength/quality [6, 45]. Furthermore, older adults are at risk for developing sarcopenic obesity (a condition characterized by low muscle mass, poor physical function/strength, and excess adiposity), which is associated with disability and high mortality [46]. Overall, lifestyle-based weight loss interventions have a positive effect on physical performance. However, findings from this study showed that unfavorable changes in body composition (i.e., increases in fat mass and decreases in lean mass) are associated with worse physical performance and weaker strength. This underscores the importance of long-term weight maintenance to prevent gain/regain of fat mass and preserve lean mass to maintain physical performance and strength in the context of lifestyle-based weight loss interventions as well as usual medical care. One strategy to promote long-term weight maintenance and to mitigate the loss of lean mass during intentional weight loss is a combination of aerobic and resistance training [8, 47], which could be particularly important to incorporate as a part of lifestyle behavior change for middle-aged and older adults with obesity and T2DM.

Notably, the effect of changes in body composition on physical function differed by age: increases in fat mass were associated with worse physical performance among those aged ≥60 but not among those aged <60 years. Excess adiposity is known to accelerate the loss of muscle mass and is associated with poor physical function in older adults [48–50], which may explain why increases in fat mass had a significant impact on physical performance in older adults compared to middle-aged adults. Additionally, the associations between decreases in lean mass and weaker strength appear to be driven by those aged <60 years.

This study has notable strengths and limitations. One strength of the study is the use of objective physical performance and strength measures. Secondly, there was high participation in the M&M ancillary study (89% of eligible participants) and a high proportion of participants (65%) with complete data for this analysis. However, the main limitation of the study is that objective measures of physical function were not measured at baseline, so we cannot assess the extent to which changes in body composition were associated with changes in physical function. Additionally, the analysis was limited to one Look AHEAD site, Baton Rouge, given its participation in both the Look AHEAD DXA sub-study and M&M ancillary study; thus, the results may not be generalizable to other Look AHEAD sites. Furthermore, results may not be generalizable to older adults with frailty or individuals without T2DM.

In conclusion, although the overall effect of intensive lifestyle intervention on physical performance was positive, increases in fat mass were associated with worse physical performance while decreases in lean mass were associated with weaker strength regardless of the intervention arm. These findings highlight the importance of preventing gain of fat mass and preserving lean mass in middle-aged and older adults with obesity and T2DM in the context of intentional weight loss and usual medical care. There are several areas of future direction to consider. Investigating the long-term, longitudinal relationship between both overall as well as regional body composition changes and *changes* in physical function will further build evidence on the long-term impact of intentional weight loss. While this study investigated the effect of lifestyle-based weight loss, the use of glucagon-like peptide-1 receptor agonists (GLP-1RAs) is gaining heightened attention due to their impressive weight loss efficacy [51]. Given the greater magnitude of weight and lean mass loss of these drugs [52], future studies should assess the effects of GLP-1RA-assisted weight loss on body composition and objective measures of physical function among middle-aged and older adults with obesity and T2DM.

## Supplementary information

Supplementary Materials

## Data Availability

The data that support the findings of this study (10.58020/wr3g-1218) are available upon request at the NIDDK Central Repository website, Resources for Research (R4R), https://repository.niddk.nih.gov/.
