# Supplementary material for: Long-term change in body composition following intentional weight loss and its effect on physical function
Source: Int J Obes (Lond). 2025 Dec 23;50(4):760–7. doi: 10.1038/s41366-025-01997-x (PMC13056564; doi:10.1038/s41366-025-01997-x)
Supplement: Supplementary file 1 — Supplementary Materials [file 41366_2025_1997_MOESM1_ESM.pdf]

Long-term Change in Body Composition Following Intentional Weight Loss and Its Effect on Physical Function

SUPPLEMENTARY MATERIALS

Table of Contents

		Page
Supplementary Material 1.	Complete listing of the Look AHEAD Movement and Memory Research Group	2
Supplementary Figure S1.	Consolidated Standards of Reporting Trials (CONSORT) diagram for participants at the Baton Rouge Look AHEAD clinic site who participated in the DXA sub study and the Look AHEAD Movement and Memory ancillary study.	3
Supplementary Table S1.	Mean differences in physical function measures by percent change in fat mass over 8 years in intensive lifestyle intervention (ILI) and diabetes support and education (DSE) participants at the Baton Rouge Look AHEAD clinic site who participated in the dual-energy X-ray absorptiometry sub-study and the Look AHEAD Movement and Memory ancillary study.	4
Supplementary Table S2.	Mean differences in physical function measures by percent change in lean mass over 8 years in intensive lifestyle intervention (ILI) and diabetes support and education (DSE) participants at the Baton Rouge Look AHEAD clinic site who participated in the dual-energy X-ray absorptiometry sub-study and the Look AHEAD Movement and Memory ancillary study	5
Supplementary Table S3.	Association between change in body composition over 8 years and physical function measures by intervention assignment and baseline age (<60 vs. ≥60 years) for participants at the Baton Rouge Look AHEAD clinic site who participated in the dual-energy X-ray absorptiometry sub-study and the Look AHEAD M&M ancillary study	6

Supplementary Material 1. Look AHEAD Movement and Memory Research Group

The authors thank all investigators and staff involved in the Look AHEAD trial. The authors acknowledge the specific clinical sites and investigators involved in the Look AHEAD Movement and Memory ancillary study.

Pennington Biomedical Research Center George A. Bray, MD[1]; Kristi Rau[2]; Allison Strate, RN[2]; Frank L. Greenway, MD[3]; Donna H. Ryan, MD[3]; Donald Williamson, PhD[3]; Brandi Armand, LPN; Jennifer Arceneaux; Amy Bachand, MA; Michelle Begnaud, LDN, RD, CDE; Betsy Berhard; Elizabeth Caderette; Barbara Cerniauskas, LDN, RD, CDE; David Creel, MA; Diane Crow; Crystal Duncan; Helen Guay, LDN, LPC, RD; Carolyn Johnson, Lisa Jones; Nancy Kora; Kelly LaFleur; Kim Landry; Missy Lingle; Jennifer Perault; Cindy Puckett; Mandy Shipp, RD; Marisa Smith; Elizabeth Tucker

University of Colorado Health Sciences Center James O. Hill, PhD[1]; Marsha Miller, MS, RD[2]; Brent Van Dorsten, PhD[3]; Judith Regensteiner, PhD[3]; Ligia Coelho, BS; Paulette Cohrs, RN, BSN; Susan Green; April Hamilton, BS, CCRC; Jere Hamilton, BA; Eugene Leshchinskiy; Lindsey Munkwitz, BS; Loretta Rome, TRS; Terra Worley, BA; Kirstie Craul, RD,CDE; Sheila Smith, BS

The University of Tennessee Health Science Center
University of Tennessee East. Karen C. Johnson, MD, MPH[1]; Carolyn Gresham, RN[2]; Stephanie Connelly, MD, MPH[3]; Amy Brewer, RD, MS; Mace Coday, PhD; Lisa Jones, RN; Lynne Lichtermann, RN, BSN; Shirley Vosburg, RD, MPH; and J. Lee Taylor, MEd, MBA. *University of Tennessee Downtown.* Abbas E. Kitabchi, PhD, MD[1]; Ebenezer Nyenwe, MD[3]; Helen Lambeth, RN, BSN[2]; Amy Brewer, MS, RD,LDN; Debra Clark, LPN; Andrea Crisler, MT; Debra Force, MS, RD, LDN; Donna Green, RN; Robert Kores, PhD

University of Pittsburgh John M. Jakicic, PhD[1], David E. Kelley, MD[1]; Jacqueline Wesche-Thobaben, RN, BSN, CDE[2]; Lewis H. Kuller, MD, DrPH[3]; Andrea Kriska, PhD[3]; Amy D. Otto, PhD, RD, LDN[3], Lin Ewing, PhD, RN[3], Mary Korytkowski, MD[3], Daniel Edmundowicz, MD[3]; Monica E. Yamamoto, DrPH, RD, FADA [3]; Rebecca Danchenko, BS; Barbara Elnyczky; David O. Garcia, MS; George A. Grove, MS; Patricia H. Harper, MS, RD, LDN; Susan Harrier, BS; Nicole L. Helbling, MS, RN; Diane Ives, MPH; Juliet Mancino, MS, RD, CDE, LDN; Anne Mathews, PhD, RD, LDN; Tracey Y. Murray, BS; Joan R. Ritchea; Susan Urda, BS, CTR; Donna L. Wolf, PhD

Coordinating Center, Wake Forest School of Medicine Stephen B. Kritchevsky, PhD[1]; Denise K. Houston, PhD[1]; Jeff D. Williamson, MD[3]; Stephen R. Rapp, PhD[3]; Mark A. Espeland, PhD[3]; Xiaoyan (Iris) Leng, MD, PhD[3]; Gary Miller, PhD[3]; Amelia Hodges, BS, CCRP[2]; Michelle Gordon, MS[2]; Jennifer Walker[2]; Tara Beckner; Jason Griffin, BS; Lea Harvin, BS; Kathy Lane, BS; Rebecca H. Neiberg, MS

---

[1] Principal Investigator
[2] Program Coordinator
[3] Co-Investigator

All other Look AHEAD staff are listed alphabetically by site.

Supplementary Figure S1: Consolidated Standards of Reporting Trials (CONSORT) diagram for participants at the Baton Rouge Look AHEAD clinic site who participated in the dual-energy X-ray absorptiometry sub study and the Look AHEAD Movement and Memory ancillary study

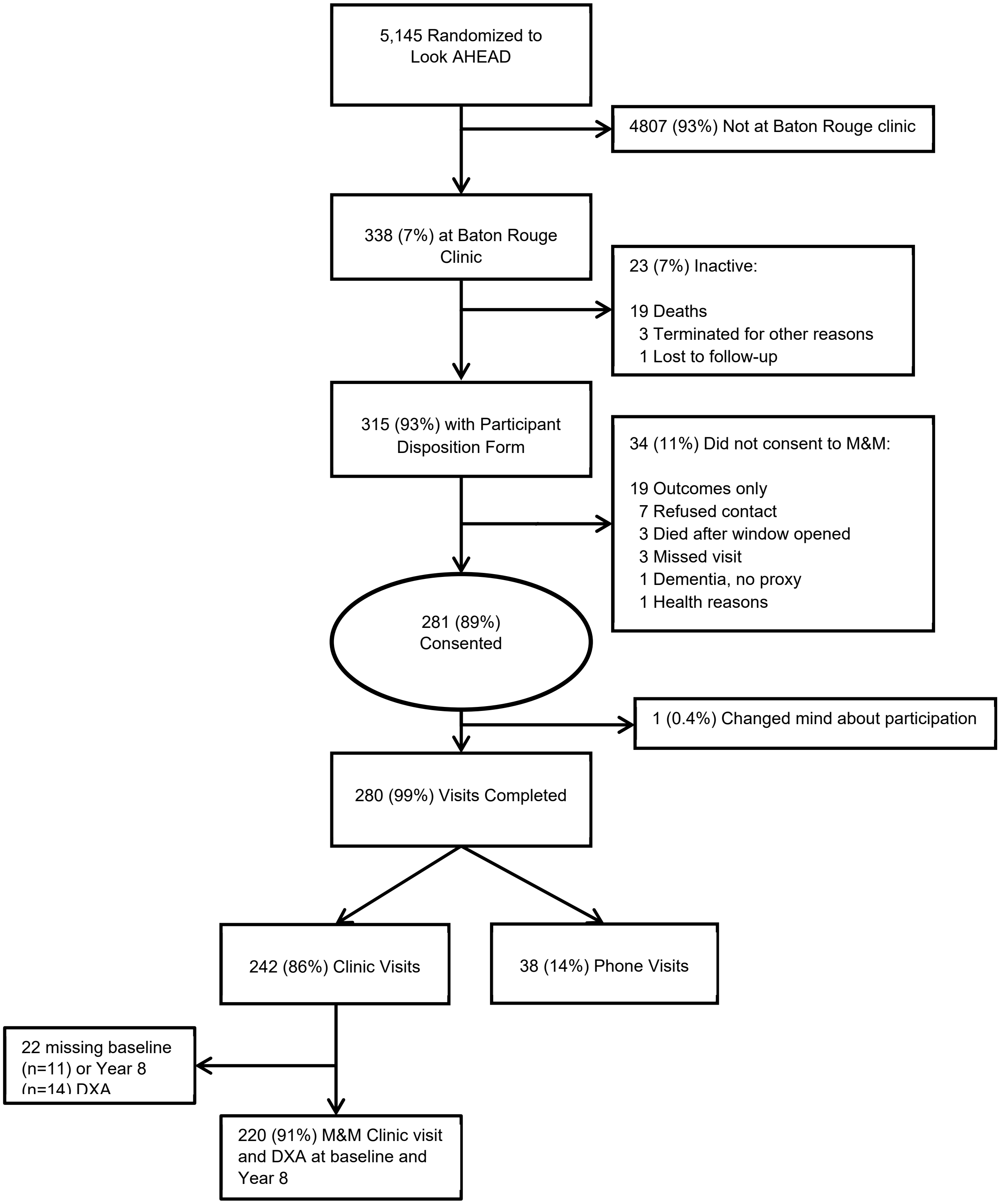

Supplementary Table S1. Mean differences in physical function measures by percent change in fat mass over 8 years in intensive lifestyle intervention (ILI) and diabetes support and education (DSE) participants at the Baton Rouge Look AHEAD clinic site who participated in the dual-energy X-ray absorptiometry sub-study and the Look AHEAD Movement and Memory ancillary study

	**Intensive Lifestyle Intervention (ILI)**		**Diabetes Support and Education (DSE)**		**ILI and DSE combined: Physical Function by intervention assignment interaction p-value**
**Physical Function Measure**	**Beta (SE) for 1% increase in % Fat Mass**	**p-value**	**Beta (SE) for 1% increase in % Fat Mass**	**p-value**	
SPPB score (range 0-12)					
Model 1[a]	0.004 (0.012)	0.74	-0.014 (0.011)	0.20	0.29
Model 2[b]	-0.003 (0.013)	0.83	-0.015 (0.014)	0.28	0.28
$SPPB_{exp}$ score (range 0-3)					
Model 1	-0.005 (0.002)	0.03	-0.004 (0.002)	0.03	0.87
Model 2	-0.006 (0.003)	0.03	-0.006 (0.002)	0.02	0.91
20-meter gait speed (m/sec)					
Model 1	-0.002 (0.001)	0.20	-0.003 (0.001)	0.01	0.61
Model 2	-0.002 (0.001)	0.19	-0.003 (0.001)	0.04	0.61
400-meter gait speed (m/sec)					
Model 1	-0.001 (0.001)	0.42	-0.002 (0.001)	0.07	0.63
Model 2	-0.002 (0.001)	0.15	-0.002 (0.001)	0.07	0.62
Grip strength (kg)					
Model 1	0.060 (0.062)	0.34	-0.009 (0.046)	0.84	0.25
Model 2	-0.001 (0.070)	0.99	-0.081 (0.055)	0.14	0.18
Knee extensor strength (maximum weight lifted; kg)					
Model 1	0.033 (0.082)	0.69	0.127 (0.064)	0.05	0.62
Model 2	-0.113 (0.091)	0.22	0.061 (0.076)	0.42	0.61

Note. Abbreviations: SPPB, Short Physical Performance Battery; $SPPB_{exp}$, Expanded Short Physical Performance Battery.

[a] Model 1: adjusted for sex, race/ethnicity, education, baseline age, BMI, HbA_{1c}, insulin use, diabetes duration, hypertension status, prior CVD, depressive symptoms, smoking, cardiorespiratory fitness, and SF-36 Physical Functioning and Bodily Pain Subscale, and year of visit.

[b] Model 2: adjusted for variables in model 1 plus percent change in lean mass.

Supplementary Table S2: Mean differences in physical function measures by percent change in lean mass over 8 years in intensive lifestyle intervention (ILI) and diabetes support and education (DSE) participants at the Baton Rouge Look AHEAD clinic site who participated in the dual-energy X-ray absorptiometry sub-study and the Look AHEAD Movement and Memory ancillary study

	**Intensive Lifestyle Intervention (ILI)**		**Diabetes Support and Education (DSE)**		**ILI and DSE combined: Physical Function by intervention assignment interaction p-value**
**Physical Function Measure**	**Beta (SE) for 1% decrease in % Lean Mass**	**p-value**	**Beta (SE) for 1% decrease in % Lean Mass**	**p-value**	
SPPB score (range 0-12)					
Model 1[a]	-0.032 (0.029)	0.27	0.021 (0.030)	0.49	0.18
Model 2[b]	-0.036 (0.033)	0.29	-0.004 (0.037)	0.92	0.21
$SPPB_{exp}$ score (range 0-3)					
Model 1	0.004 (0.006)	0.56	0.002 (0.005)	0.76	0.69
Model 2	-0.004 (0.007)	0.58	-0.007 (0.006)	0.23	0.89
20-meter gait speed (m/sec)					
Model 1	0.001 (0.003)	0.82	0.005 (0.003)	0.09	0.17
Model 2	-0.001 (0.003)	0.67	0.000 (0.003)	0.92	0.25
400-meter gait speed (m/sec)					
Model 1	0.002 (0.003)	0.44	0.002 (0.003)	0.53	0.16
Model 2	-0.005 (0.003)	0.15	-0.002 (0.003)	0.54	0.20
Grip strength (kg)					
Model 1	-0.325 (0.153)	0.04	-0.199 (0.116)	0.09	0.57
Model 2	-0.326 (0.175)	0.07	-0.321 (0.142)	0.03	0.61
Knee extensor strength (maximum weight lifted; kg)					
Model 1	-0.495 (0.179)	<0.01	-0.432 (0.179)	0.02	0.28
Model 2	-0.633 (0.210)	<0.01	-0.337 (0.215)	0.12	0.29

Note. Abbreviations: SPPB, Short Physical Performance Battery; $SPPB_{exp}$, Expanded Short Physical Performance Battery.

[a] Model 1: adjusted for sex, race/ethnicity, education, baseline age, BMI, HbA_{1c}, insulin use, diabetes duration, hypertension status, prior CVD, depressive symptoms, smoking, cardiorespiratory fitness, and SF-36 Physical Functioning and Bodily Pain Subscale, and year of visit.

[b] Model 2: adjusted for variables in model 1 plus percent change in fat mass.

Supplementary Table S3: Association between change in body composition over 8 years and physical function measures by intervention assignment and baseline age (<60 vs. ≥60 years) for participants at the Baton Rouge Look AHEAD clinic site who participated in the dual-energy X-ray absorptiometry sub-study and the Look AHEAD M&M ancillary study

	Intensive Lifestyle Intervention (ILI)			Diabetes Support and Education (DSE)		
	Age <60 yrs	Age ≥60 yrs	Interaction p-value	Age <60 yrs	Age ≥60 yrs	Interaction p-value
**Physical Function Measure**	Beta (SE)[b]	Beta (SE)		Beta (SE)	Beta (SE)	
**% Change in fat mass**						
SPPB score (range 0-12)	0.013 (0.013)	-0.036 (0.023)	0.04	-0.007 (0.016)	-0.026 (0.023)	0.99
$SPPB_{exp}$ score (range 0-3)	-0.002 (0.002)	-0.017 (0.005)	0.001	-0.003 (0.003)	-0.008 (0.004)	0.66
20-meter gait speed (m/sec)	-0.000 (0.002)	-0.006 (0.002)	0.006	-0.002 (0.001)	-0.002 (0.002)	0.91
400-meter gait speed (m/sec)	-0.001 (0.001)	-0.003 (0.003)	0.30	-0.001 (0.002)	-0.002 (0.002)	0.67
Grip strength (kg)	0.033 (0.079)	0.120 (0.114)	0.66	0.019 (0.057)	-0.114 (0.110)	0.40
Knee extensor strength (maximum weight lifted; kg)	0.037 (0.117)	-0.026 (0.152)	0.45	0.118 (0.116)	0.113 (0.109)	0.97
**% Change in lean mass**						
SPPB score (range 0-12)	-0.004 (0.035)	0.028 (0.059)	0.45	-0.031 (0.048)	0.041 (0.054)	0.35
$SPPB_{exp}$ score (range 0-3)	0.004 (0.005)	0.014 (0.014)	0.62	-0.008 (0.008)	0.006 (0.009)	0.23
20-meter gait speed (m/sec)	0.002 (0.004)	0.002 (0.006)	0.94	0.003 (0.004)	0.003 (0.005)	0.71
400-meter gait speed (m/sec)	-0.003 (0.004)	-0.006 (0.007)	0.98	-0.003 (0.005)	0.005 (0.005)	0.44
Grip strength (kg)	0.003 (0.209)	-0.637 (0.254)	0.47	-0.326 (0.150)	0.039 (0.267)	0.44
Knee extensor strength (maximum weight lifted; kg)	-0.666 (0.239)	-0.303 (0.378)	0.17	-0.940 (0.246)	-0.226 (0.411)	0.03

Note. Abbreviations: SPPB, Short Physical Performance Battery; $SPPB_{exp}$, Expanded Short Physical Performance Battery.

[a] Adjusted for sex, race/ethnicity, education, year of visit, and baseline BMI, HbA_{1c}, insulin use, diabetes duration, hypertension status, prior CVD, depressive symptoms, smoking, cardiorespiratory fitness, and SF-36 Physical Functioning and Bodily Pain subscales.

[b] Beta (SE) for every 1% increase in fat mass or 1% decrease in lean mass.
